# Exploring Viral Diversity in a Gypsum Karst Lake Ecosystem Using Targeted Single-Cell Genomics

**DOI:** 10.3390/genes12060886

**Published:** 2021-06-08

**Authors:** Sigitas Šulčius, Gediminas Alzbutas, Viktorija Juknevičiūtė, Eugenijus Šimoliūnas, Petras Venckus, Monika Šimoliūnienė, Ričardas Paškauskas

**Affiliations:** 1 Laboratory of Algology and Microbial Ecology, Nature Research Centre, Akademijos Str. 2, LT-08412 Vilnius, Lithuania; gediminas.alzbutas@gamtc.lt (G.A.); jukne.viktorija@gmail.com (V.J.); petras.venckus@gamtc.lt (P.V.); ricardas.paskauskas@gamtc.lt (R.P.); 2 Department of Molecular Microbiology and Biotechnology, Institute of Biochemistry, Life Sciences Centre, Vilnius University, Saulėtekio Av. 7, LT-10257 Vilnius, Lithuania; eugenijus.simoliunas@bchi.vu.lt (E.Š.); monika.simoliuniene@gmc.vu.lt (M.Š.)

**Keywords:** chlorobium phages, green sulfur bacteria, gypsum karst ecosystem, Kirkilai, Lithuania, Microviridae, Natura 2000, rare ecosystems, virus diversity, Single Cell Genomics

## Abstract

Little is known about the diversity and distribution of viruses infecting green sulfur bacteria (GSB) thriving in euxinic (sulfuric and anoxic) habitats, including gypsum karst lake ecosystems. In this study, we used targeted cell sorting combined with single-cell sequencing to gain insights into the gene content and genomic potential of viruses infecting sulfur-oxidizing bacteria *Chlorobium clathratiforme*, obtained from water samples collected during summer stratification in gypsum karst Lake Kirkilai (Lithuania). In total, 82 viral contigs were bioinformatically identified in 62 single amplified genomes (SAGs) of *C. clathratiforme*. The majority of viral gene and protein sequences showed little to no similarity with phage sequences in public databases, uncovering the vast diversity of previously undescribed GSB viruses. We observed a high level of lysogenization in the *C. clathratiforme* population, as 87% SAGs contained intact prophages. Among the thirty identified auxiliary metabolic genes (AMGs), two, thiosulfate sulfurtransferase (TST) and thioredoxin-dependent phosphoadenosine phosphosulfate (PAPS) reductase (cysH), were found to be involved in the oxidation of inorganic sulfur compounds, suggesting that viruses can influence the metabolism and cycling of this essential element. Finally, the analysis of CRISPR spacers retrieved from the consensus *C. clathratiforme* genome imply persistent and active virus–host interactions for several putative phages prevalent among *C. clathratiforme* SAGs. Overall, this study provides a glimpse into the diversity of phages associated with naturally occurring and highly abundant sulfur-oxidizing bacteria.

## 1. Introduction

Lakes of gypsum karst are small, yet very complex, aquatic ecosystems formed in sinkholes that emerge due to the erosion of gypsiferous dolomite bedrock. These endemic lakes can be characterized by strong thermal and chemical stratification, leading to contrasting gradients in redox potential and microbial metabolism [1,2]. Generally, aerobic oxygenic photolithoautotrophic processes dominate the surface waters of stratified lakes. In contrast, anaerobic anoxygenic photo/chemolithotrophic or heterotrophic processes (e.g., mediated by sulfur-oxidizing and sulfate-reducing bacteria) prevail in the euxinic (sulfuric and anoxic) layers of the water column [3,4,5]. Therefore, it might also be expected that bacterial communities in karstic lakes will exhibit pronounced vertical differences in taxonomic composition and gene content, corresponding to their physiological requirements and taxon-specific biogeochemical functions [1]. Although information about microbial diversity across the vertical gradients in (gypsum) karstic lake ecosystems remains scattered and fragmented (with very few sequencing datasets available in current public repositories), the available studies document the strong selective pressure of environmental factors on individual taxa in several karstic and stratified lakes [6,7,8,9]. In contrast, the role of the biotic factors, and especially of (bacterio)phages, remains poorly comprehended, leading to an insufficient understanding of the food web dynamics in these ecosystems. Consequently, there is a strong need to explore the diversity and genomic potential of phages in the context of the prevailing microbial assemblages throughout the environmental gradients of the karst lake ecosystems, and, more importantly, to identify the existing links between different members of viral and bacterial communities.

Phages are the most numerous and diverse component of the aquatic microbial food web. Phages can modulate cellular metabolism and regulate host population size, and through that, influence the biogeochemical cycles of essential elements (e.g., carbon, nitrogen, etc. [10,11]). They can mediate gene transfer between microorganisms and, therefore, contribute to species diversification and evolution. Currently, aquatic viromics (that is, metagenomics of the sample size fraction passing through 0.2 µm filters) is the most commonly used approach to characterize the composition and structure of uncultured viral communities in the environments [12]. Although it enables the *en masse* sequencing of entire viral assemblages and to some degree provides quantitative community assessment, the taxonomic assignments and phylogenomic analyses are limited by the relatively short-read assemblies and the lack of reference viral genomes in public databases [13]. Alternatively, the sequencing of individual virions [14,15,16] or viral DNA cloned into fosmids [17,18] enables us to obtain much larger DNA fragments, and even the entire genomes, from the environmental samples, although these approaches can either over- or underrepresent the most common members in the viral community [15,19]. In any case, the approaches mentioned above have mainly been used so far to represent a diversity of free viruses (in opposition to intracellular phages), implying that the host cells they have been infecting are already lysed and thus absent from the bacterial assemblages. In turn, this makes it difficult to establish links between the co-occurring viral and bacterial communities [13]. To overcome this issue and to establish possible associations between phages and bacteria, the single-cell sequencing and bioinformatic reconstruction of viral reads from within the single amplified genomes (SAGs) of the infected cells can be used [16,20,21,22,23]. If successful, the mining of SAGs datasets not only makes possible the discovery of novel phage genomes [24], but, more importantly, elucidates the ecological relevance and enables a more robust classification of viral sequences [23].

Lake Kirkilai (56°14′55.5″ N, 24°41′33.7″ E; Biržai, Lithuania) represents a rare habitat of gypsum karst lakes (Natura 2000 code 3190), which are distinguished from other karstic ecosystems of a sinkhole origin (e.g., turloughs; Natura 2000 code 3180) by the permanent presence of water (EUR 28). This feature allows the formation of relatively stable hydrological and habitat connectivity across spatiotemporal scales, with potentially more resilient microbial communities than those in temporal karstic lakes [25,26]. Lake Kirkilai is a shallow (max. depth 6–7 m), nutrient- and organic matter-rich water body with high concentrations of Ca^2+^ and SO_4_^2−^ ions [27]. During summer stratification, the lake exhibits a pronounced chemocline at approximately 2 m deep, which is noticeable due to the depletion of oxygen, changes in water conductivity, and the presence of hydrogen sulfide (H_2_S) [28,29,30]. These conditions are also accompanied by a significant shift in water temperature and density. Previous studies based on pigment analysis and partial 16S rRNA gene sequences obtained from the enrichment cultures revealed that members of genera *Chlorobium* (green sulfur-oxidizing bacteria; GSB) and *Desulfovibrio* (sulfate-reducing bacterium; SRB) are prevalent in the euxinic water layers of Lake Kirkilai [28,29,30]. At present, however, the relative abundance and distribution of these species, and any associations with other members of the lake bacterial or viral communities, are unknown.

This study aimed to explore the diversity of bacteriophages associated with the most common (keystone) taxa of the bacterial community in the Lake Kirkilai, a model system of the unique gypsum karst environment. Toward this aim, we first assessed the structure and distribution of the microbial assemblages across the vertical gradient of the water column using 16S rRNA gene amplicon sequencing. Next, we applied targeted cell sorting to isolate and sequence individual cells of the most abundant taxa found in the euxinic water layers, a sulfur-oxidizing bacterium closely related to *C. clathratiforme* (formerly *Pelodictyon phaeoclathratiforme*; [31]). We were able to recover 82 viral contigs from 62 single amplified genomes (SAGs) of *C. clathratiforme* representing partial (74/84), near-complete (5/82), or complete (3/82) dsDNA and ssDNA (1 genome) viral genomes, including both lytic and temperate phages, putatively infecting members of the *C. clathratiforme* population. The identified viral sequences showed little or no similarity to other known phage sequences in public databases, nor were they similar to each other, suggesting the vast and largely overlooked diversity of phages associated with green sulfur bacteria. Some phages encoded auxiliary metabolic genes (AMGs) involved in sulfur metabolism, indicating an intervening role of viruses in the biogeochemical cycling of this element. By co-assembling and binning multiple and closely related *C. clathratiforme* SAGs, we reconstructed a near-complete (99%) consensus genome of *C. clathratiforme*. Analysis of *C. clathratiforme* CRISPR arrays provides a glimpse into the dynamic interactions between *C. clathratiforme* and the putative phages identified in this study.

## 2. Materials and Methods

Water samples were collected via a hand-made submersible water collection device connected to an on-boat peristaltic pump. Water intake vents were set up every 0.5 m from surface to 5 m depth. The device was carefully immersed in water and left to settle for several hours before the sampling. This approach allowed us to collect water at the precise depth without intermixing different layers of the stratified water column.

Samples for 16S amplicon sequencing were collected at 1 m, 3 m, 4 m, and 5 m depths. At each depth, 10 L of lake water was first pre-filtered through 10 µm Polycap AS filters (Millipore) and then collected (1–3 L) in Sterivex filter units (0.22 µm, Merck KGaA, Darmstadt, Germany) using a peristaltic pump. Sterivex filter units were filled with 1.8 mL of lysis buffer (40 mM EDTA, 50 mM Tris-HCL), flash-frozen in liquid nitrogen, and stored at −80 °C until further processing. DNA was extracted using the phenol/chloroform approach according to descriptions in Riemann et al. [32]. For 16S rRNA gene fragment amplification, we used 515F-Y/926R primers [33], which target the V4-V5 region of the 16S rRNA gene. Polymerase chain reactions (PCR) were carried out using 2× Phusion Master Mix with HF Buffer (ThermoFisher Scientific Baltics, Vilnius, Lithuania) and 1 µL of genomic DNA as the template. The PCR cycling conditions included an initial 30 s denaturing step at 98 °C followed by 30 cycles of denaturation at 98 °C for 15 s, annealing at 51 °C for 45 s and elongation at 72 °C for 45 s, and a final elongation step at 72 °C for 90 s. PCR fragments were excised from 2% agarose gels and purified with the GeneJET™ Gel Extraction and DNA Cleanup Micro Kit (ThermoFisher Scientific Baltics, Vilnius, Lithuania). DNA libraries were generated using the Collibri™ PS DNA Library Prep Kit for Illumina Systems (ThermoFisher Scientific Baltics, Vilnius, Lithuania), and were normalized and pooled together before sequencing. Sequencing was conducted on an Illumina MiSeq platform using 2 × 300 bp paired-end reads based on the protocol at the ThermoFisher Scientific Baltics (Vilnius, Lithuania).

Samples for single-cell sequencing were collected at 3.5 m depth and processed as described in the SCGC protocol (scgc.bigelow.org (accessed on 17 July 2020)). Single-cell sorting, whole-genome amplification (WGA-X; multiple displacement amplification (MDA) of DNA that utilizes a thermostable mutant of the phi29 polymerase), and real-time PCR screens were performed at the Bigelow Laboratory Single Cell Genomics Center (bigelow.org/scgc (accessed on 10 November 2020)). The sorting gates for the targeted sorting of *Chlorobium* bacteria were defined based on the autofluorescence of the characteristic cellular bacteriochlorophylls *c*, *d,* and *e* (absorption maxima 715–745 nm). Based on the effective WGA-X amplification of genetic material, a 384-well plate was selected for low-coverage shotgun sequencing with an Illumina end-paired HiSeq.

The 16S rDNA amplicon sequences were trimmed of adapters and quality-checked using the bbduk available in BBMap 37.17 package (jgi.doe.gov/data-and-tools/bb-tools/ (accessed on 10 December 2020)) with the following settings: ktrim = r; k = 23; mink = 11; hdist = 1; minlength = 50; maxns = 1; qtrim = r; trimq = 15 tpe tbo. Singletons and chimeric sequences were removed before further processing of the sequence reads. Sequences in each sample were randomly subsampled using seqkit v0.7.0 [34] to 100,000 read pairs so that all samples had the same number of sequences (equal to the sample with the fewest total sequences). For the OTU analysis, the reads were initially merged using bbmerge from the BBMap 37.17 package. The de-replication was conducted using vsearch v2.14.2 [35] followed by clustering with swarm v3.0.0 [36] with the parameter d set to 2. The taxonomic assignment of OTUs was conducted using a naïve Bayesian classifier from DADA2 (with minBoot) using the SILVA v138 database as a reference [37]. Sequence counts and taxonomic assignments of the most abundant OTUs (>0.1% from the total reads) were compiled in R and visualized using the phyloseq package v3.10 [38].

The obtained reads of SAGs were trimmed, normalized with kmernorm (https://sourceforge.net/projects/kmernorm/ (accessed on 10 December 2020)), and assembled with SPAdes version: 3.13.1 [39]. The CheckM v1.0.12 [40] was used to estimate the genome size and completeness of SAGs. Then, contigs longer than 20 kb were merged into a single dataset and reassembled with SPAdes using the command-line option “--only assembler”. In addition, assembly reads were merged and aligned using BBMap version 38.84 (sourceforge.net/projects/bbmap/ (accessed on 10 December 2020)), normalized using BBNorm with non-default command-line options “target = 100 min = 1 fixspikes = t” and quality trimmed using BBDuk with non-default command-line options “ktrim = r k = 23 mink = 11 hdist = 1 tpe tbo qtrim = r trimq = 15 threads = 12”. The overlapping reads were merged using BBMerge with default settings. The resulting contigs were once again reassembled with SPAdes. Viral sequences were identified using Vibrant v1.2.1 [41] and classified using Kraken2 [42] (viral DB version of 12/2/2020; benlangmead.github.io/aws-indexes/k2 (accessed on 2 January 2021)) and Kaiju [43] (viral DB version of 2020-05-25; kaiju.binf.ku.dk/server (accessed on 2 January 2021)). Distribution of identified viral contigs was assessed using BLASTn search against IMG/VR dataset (Ecosystem category: Aquatic; accessed June 2021; database version 5.1) with the average nucleotide identity of ≥95% over the alignment length of ≥85%, and E-value ≤ 1 considered as significant.

The consensus genome of *C. clathratiforme* was assembled using the MetaWRAP 1.3 bin refinement module [44] after the binning of contigs by MaxBin2 v2.2.6 [45], Concoot v1.0.0 [46], MetaBAT1 from MetaBAT2 v2.12.1 [47]. Prior to the binning procedure, *C. clathratiforme* contigs were classified using Kraken2 (“Standard” DB) and Kaiju (“nr” DB). Genome quality and completeness were evaluated using CheckM. The genomes were automatically annotated using PROKKA v1.14.5 [48]. CRISPR-Cas genes and arrays in the assembled consensus *C. clathratiforme* genome were identified using CRISPRCasTyper v 1.2.4 [49] and CRISPRidentify v 1.0.0 [50]. SpacePHARER v4.228b9e5 [51] was used to detect matches against viral contigs detected in SAGs and against GenBank_phage_2018_09 DB available within SpacePHARER.

Temperature, salinity, conductivity, and dissolved oxygen concentration were measured in situ using a multi-parameter portable meter MultiLine F/Set-3 (WTW). Nutrient concentrations, including nitrate, nitrite, and phosphate, were determined in a certified laboratory following standard procedures (ISO 7150-1, ISO13395-2000, ISO 11905-1:1997, and ISO 15681-1:2005).

## 3. Results

### 3.1. Distribution of Bacterial Community throughout the Water Column of Lake Kirkilai

**Figure 1:**
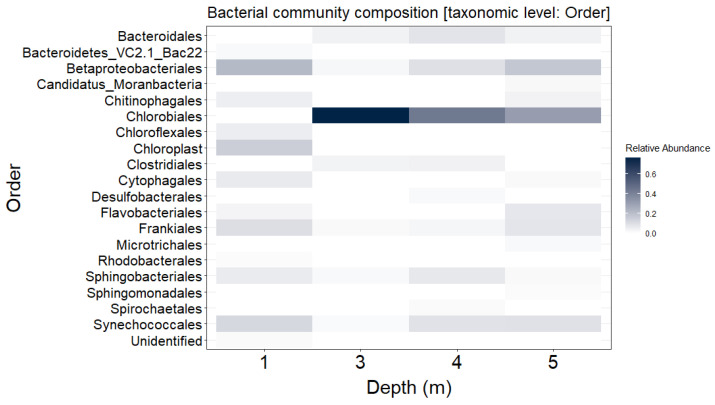
Bacterial community composition of the most abundant (>1% from total reads) taxa throughout the water column of the gypsum karst Lake Kirkilai (Lithuania).

Bacterial community composition in Lake Kirkilai was examined by 16S rRNA gene amplicon sequencing of water samples collected at four different depths: at the surface (1 m) and in the euxinic zone at 3 m, 4 m, and 5 m depth; predefined by the in situ measurements of environmental variables (Table S1). In total, 3479 OTUs were identified by swarm v3.0.0 [36], with 267 singletons (7.7%) and 1046 doubletons (30%). Rarefaction curves reached a near plateau for samples taken at 1 m and 3 m depths (Figure S1), suggesting that these datasets can be considered sufficient to assess bacterial diversity. The rarefaction curve for samples taken at 4 m and 5 m depths did not reach a plateau (Figure S1), indicating that the diversity in these samples might be underestimated. Nevertheless, the calculated Shannon and Simpson diversity indices indicated moderate-to-high microbial diversity at all depths (Figure S2), except for the sample taken at 3 m depth, dominated by a single taxon (Figure 1; Table S2; also see below).

A total of nine bacterial phyla comprising 1% or more of the total reads were identified (Table S2). Cyanobacteria were the dominant (33%) phylum in the surface water layer, accompanied by major co-existing bacterial phyla such as *Bacteroidetes* (24%), *Proteobacteria* (22%) and *Actinobacteria* (12%). In the euxinic water zone, members of order *Chlorobiales* were the most abundant, comprising up to 76% of the total bacterial abundance (Figure 1). Within *Chlorobiales*, most of the sequences were grouped into a single OTU (OTU2; Table S2) closely related to *Chlorobium* sp. This OUT was three orders of magnitude more abundant than the second-ranked OTU at the same depth (Table S2). The proportion of OTUs shared between the surface (1 m, oxygenated water layer) and the euxinic water samples was 54%, with the highest values (68%) observed between 1 m and 5 m depths. The percentage of abundant OTUs (comprising ≥1% of total reads) shared between the samples taken from within the euxinic zone (3 m–5 m; Table S1) was lower (18%), suggesting stratification of the bacterial communities. The proportion of unclassified bacterial sequences, most of which were considered rare (e.g., accounted for less than 1% from the total reads), varied from 13% to 24%, which may indicate either undersampled (e.g., at 4 m and 5 m depths; Figure S1) or still undiscovered diversity in the gypsum karst lake microbial assemblages.

### 3.2. Bacterial Diversity within Single Amplified Genomes

A total of 320 cells discriminated by size and fluorescence were sorted and subjected to MDA. The CheckM algorithm, which relies on the use of lineage-specific conserved marker genes, taxonomically classified 84 SAGs, of which 62 were identified as closely related to *C. clathratiforme* (Imhoff 2003, 2014; homotypic synonym of *P*. *phaeoclathratiforme* BU-1; Table S3, Figure S3), photoautotrophic reduced-sulfur compounds utilizing bacteria. The size of *C. clathratiforme* SAGs ranged between 24.5 kb and 1.55 Mb (average size of 0.6 Mb ± 44.3 kb), with the estimated genome completeness varying between 1% and 50% (average 18.1 ± 10.9%). All *C. clathratiforme* SAGs from which 16S rRNA gene sequences were recovered fell into a single OTU based on clustering at the 99% identity threshold, indicating the clonal sequence diversity of the population. The remaining classified SAGs belonged to 11 different taxonomic orders (Table S3), mainly represented by a single SAG, similar to those observed by 16S amplicon sequencing at 3 m and 4 m depths (Table S2). A large fraction of SAGs (236/320) were left unclassified due to the small size of the assembled genomes and the insufficient number of either taxon-specific marker genes or short sequence length of 16S rDNA (Table S3). All but *C. clathratiforme* SAGs were discarded from subsequent analyses.

### 3.3. Diversity and Distribution of Chlorobium clathratiforme-Associated Phage Sequences

**Figure 2:**
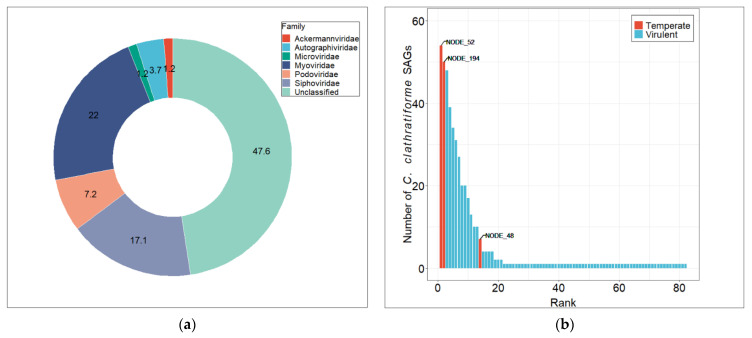
Taxonomic (Kaiju) classification (**a**) and distribution (**b**) of viral contigs recovered from 62 *C. clathratiforme* single amplified genomes (SAGs). Phage contigs were ordered by detection frequency among 62 *C. clathratiforme* SAGs.

In total, 82 viral contigs were identified in 62 *C. clathratiforme* SAGs using VIBRANT, a protein similarity scoring-based approach enabling differentiation between viral and cellular sequences [41]. The size of the viral contigs ranged from 1.4 kb to 53.5 kb, representing 3 complete, 5 near-complete (high quality) and 76 partial (low quality) genome sequences (Table S4). Further taxonomic classification of viral contigs was performed using Kaiju [43] and showed that among all classified viral contigs, members of *Myoviridae* (22%) were dominating the viral community, followed by *Siphoviridae* (17%) and *Podoviridae* (7%) (Figure 2a). A large fraction of viral contigs (~48%) was left taxonomically unassigned by the Kaiju algorithm (Figure 2a), although this proportion may change significantly if alternative classification methods are used (e.g., Kraken2; for details see Table S4). To assess the overall diversity of the viral contigs recovered from *C. clathratiforme* SAGs and to compare those sequences to publicly available genome databases, we used whole-genome-wide protein sequence similarity analysis available with ViPTree software [52]. We observed an almost complete lack of detectable genomic sequence similarities (S_G_ < 0.05; S_G_ = 1 when genomes are identical and S_G_ = 0 when a tBLASTx analysis fails to detect any sequence similarities [52]) between viral contigs and the genomes in public repositories (Figure S4). Most of the viral contigs clustered by themselves (as singletons), which thus represented a significant fraction of the previously undiscovered diversity of viral gene and protein sequences. These findings were also consistent with the BLASTn search of viral contigs against the IMG/VR aquatic dataset (Table S5). Although some of the viral contigs had matches in various metagenomes (Table S5), none of them passed the suggested standard threshold of 95% average nucleotide identity over 85% alignment fraction [53].

In total, 61 out of 62 *C. clathratiforme* SAGs contained at least 1 viral contig (Table S6), and up to 38 unique viral contigs were found in an individual SAG (SAG AH-978-L18; Table S6). The relative abundances of these contigs varied between the SAGs (Table S6), indicating extensive and rather individual cell-specific past and ongoing interactions between these bacterial and viral communities. Further, to assess the distribution of viral contigs within the *C. clathratiforme* population, we used fragment recruitment analysis against *C. clathratiforme* SAGs. This detection frequency analysis showed that the *C. clathratiforme* SAGs-associated viral community exhibited rank-abundance distribution (Figure 2b), reflecting the Bank model proposed by Breitbart and Rohwer [54]. In total, 14 viral contigs were found in more than 10% (six cells) of *C. clathratiforme* SAGs, while most of the other contigs were present only in individual cells (Figure 2b). Two contigs of putative viral genomes (24.5 kb and 8.9 kb), predicted to be temperate phages, were found in 87.1% (54/62; NODE_52; identified as integrated prophage by Vibrant software) and 80.6% (50/62; NODE_194) of the *C. clathratiforme* SAGs, suggesting a high frequency of lysogenization within the *C. clathratiforme* population. One of these putative prophages (NODE_25; Table S4) can also be found in the type strain *P. phaeoclathratiforme* BU-1 genome, a green sulfur bacterium isolated from the meromictic lake in Germany [55], which indicates the non-endemic distribution of this temperate bacteriophage. The third putative prophage (NODE_48; Table S4) was found in 7 (11.3%) out of 62 *C. clathratiforme* SAGs. Among the potentially lytic phage contigs, four (NODE_236, NODE_345, NODE_453 and NODE_232) were found in more than half of the analyzed SAGs (Table S4).

**Figure 3:**
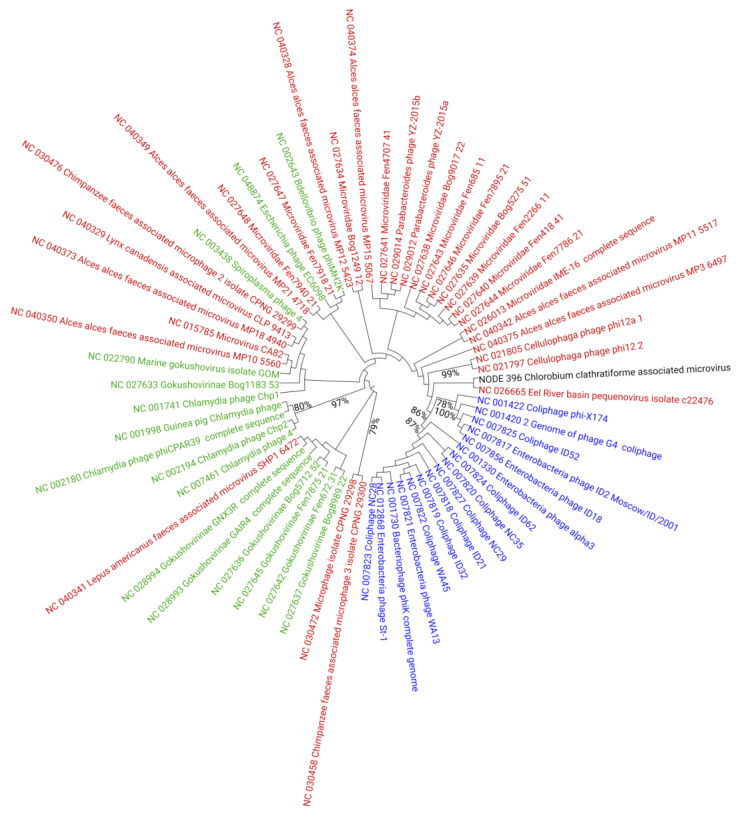
Phylogenetic tree reconstruction of *Microviridae* family bacteriophages (RefSeq DB; accessed on March 2021) and representation of *C. clathratiforme* SAGs-associated *Microviridae* genome (NODE_396). Members of *Bullavirinae* are marked in blue, members of *Gokushovirinae* are marked in green and unclassified microviruses are marked in red. The *C. clathratiforme* SAGs-associated *Microviridae* phage genome (NODE_396) is shown in black. The tree was calculated using a neighbor joining algorithm of the conserved sites in the genomes, with the bootstrap values higher than 75% given at the nodes.

To further evaluate the diversity of *C. clathratiforme* SAGs-associated viruses, we focused on eight medium- and high-quality viral contigs, including two complete and five draft genomes classified as members of *Caudovirales* (Table S4), and one complete genome of the *Microviridae* family phage. The genome annotations of these viral contigs revealed that the overall genome organizations were in general similar to those observed in other bacteriophages belonging to different families of *Caudovirales* (Table S7). At the same time, both genomic alignments (Figure S4) and single gene phylogenies (Figure S5) performed at the amino acid sequence level indicated that the *C. clathratiforme* SAGs-associated viral contigs observed in this study share little sequence conservation with viral genomes in public databases and, therefore, represented potentially new viral species, and in most cases likely new genera as well. The phylogenomic reconstruction (neighbor-joining method after alignment with MAFFT [56]) based only on the conserved genome sites of 1 *Microviridae* genome (NODE_396) recovered from *C. clathratiforme* SAGs and 62 reference *Microviridae* genomes (RefSeq viral DB; accessed in March 2021) showed that the *C. clathratiforme* SAGs-associated *Microviridae* genome (NODE_396), although grouped with *Bullavirinae* by the used algorithm, clustered distantly from other known *Microviridae* phages within the tree (Figure 3), indicating little similarity to these phages. The complete genome alignments using VIRIDIC [57], however, demonstrated slightly higher similarities between the *C. clathratiforme* SAGs-associated *Microviridae* genome (NODE_396) and *Microviridae* genomes retrieved from RefSeq DB (Table S8). For example, the *Microviridae* genome (NC_028994), reconstructed from viromes collected in Boiling Springs Lake (an acidic fumarole-heated lake) in the USA, showed 51.7% nucleotide sequence similarity at the whole-genome level (Table S8), suggesting that the two viruses may belong to the same genera [58].

### 3.4. Gene Content Analysis of Chlorobium clathratiforme-Associated Phage Sequences

In total, 1261 predicted open reading frames (ORFs) were recovered from 82 *C. clathratiforme* SAGs-associated viral contigs (Table S9). A large fraction of ORFs (855, or 68% of the total number of ORFs) were considered to be hypothetical proteins. At the same time, most of the identified phage ORFs possessed functions of viral DNA replication (e.g., helicases, primases, polymerases, etc.) and packaging enzymes (e.g., terminases), virion morphogenesis and structural genes (portal, minor and major capsid, tail tape measure, baseplate, etc.), as well as those required for cell entry (e.g., cell wall-associated hydrolase) and cell wall lysis (e.g., lysozyme, holin, etc.). The other identified ORFs were encoding proteins involved in virus–host interactions and various cell metabolic processes, including host signaling, methylation, recombination, nutrient cycling, antibiotic resistance, and transmembrane transfer of different molecules (Table S9). Thirty putative auxiliary metabolic genes (AMGs) were detected in viral contigs (Table 1), including those involved in amino acid, carbohydrate, and secondary metabolite metabolism. Most of these AMGs are commonly found among other marine and freshwater bacteriophage genomes (e.g., [59]). More intriguingly, two AMGs, *TST* and *cysH*, encoding, respectively, thiosulfate sulfurtransferase and thioredoxin-dependent phosphoadenosine phosphosulfate (PAPS) reductase, associated with sulfur metabolism were found in *C. clathratiforme* SAGs-associated viral contigs (Table 1). The BLASTp search of these AMGs against the *Chlorobium*/*Pelodictyon* database (NCBI taxid:274493) and *C. clathratiforme* SAGs revealed no significant hits, suggesting early differentiation of these genes throughout the co-evolutionary history of *C. clathratiforme* and its putative phages. A putative phosphate transport regulatory protein, which was hypothesized to be involved in phosphate scavenging and oxidative stress response, encoded by *phoU* was also identified (Table 1). Finally, four viral contigs were found to encode ribosomal proteins (L1, L18 and S10) and one ribosome-associated GTPase (Table S9). These genes were recently shown to be widespread among aquatic phages and were proposed to be involved in host metabolism takeover during infection [60]. The discovery of these AMGs broadens the known functional gene repertoires and metabolic potentials of aquatic viruses.

### 3.5. Analysis of CRISPR-Cas Loci in the Chlorobium clathratiforme Genome

By pooling and co-assembling reads from 62 *C. clathratiforme* SAGs, we reconstructed a consensus genome of *C. clathratiforme* (Supplementary File S1). The estimated completeness of the whole-genome assembly was 99%, and the resulting chromosome was 4.0 Mbp in length. In total, 3831 protein-coding genes, 2283 of which were annotated encoding hypothetical proteins, were identified using PROKKA software [48]. The assembled genome showed 98% average nucleotide identity to the type strain *P.phaeoclathratiforme* BU-1. Three CRISPR arrays (CRISPR1-3) were found in the assembled *C. clathratiforme* consensus genome (Table 2) and were based on the presence and position of the specific cas genes. They were assigned to subtypes III-A (CRISPR1), I-C (CRISPR2) and unknown (CRISPR3; contained only cas1 and cas2). All CRISPR arrays were associated with specific identical repeat sequences, while only subtype III-A appeared to have fully functional cas gene clusters with modules for spacer insertion (cas1/cas2) and target interference (various cas, csm, cmr genes; Table 2). In total, CRISPR arrays harbored 152 (CRISPR1—40, CRISPR2—31, CRISPR3—81) different spacers (Table S10), most of which were unique. A SpacePHARER search using all observed CRISPR spacers as a query found 14 significant hits to both *C. clathratiforme* SAGs-associated viral contigs observed in this study and viral genome sequences available in public databases (viralDB; accessed in March 2021; Table 2). Five different spacers from all three CRISPR arrays were found to target the same viral contig (NODE_31; Table 2), though different regions within the viral genome (all but one encoding hypothetical proteins). The positions of these spacers within the CRISPR arrays varied from the leader end (e.g., CRISPR2:1; Table 2), representing the most recent acquisition, to the middle-trailer end (e.g., CRISPR3:66; Table 2), where the oldest spacers in the locus reside. Fewer spacers, yet similarly distributed along the CRISPR arrays, were also found against the other two abundant viral contigs (NODE_113 and NODE_48; both contigs were found in more than 10% of *C. clathratiforme* SAGs; Table S4), indicating previous and ongoing co-evolution between these viruses and their hosts. Two spacers were found to have matches in *C. clathratiforme* SAGs-associated Microviridae genomes (NODE_396; CRISPR2:5) and in *Microviridae* sp. isolate ctec913 (CRISPR2:16), which was recovered from animal viromes [61].

## 4. Discussion

The knowledge about the diversity and distribution of viruses infecting green sulfur bacteria (GSB) is still extremely sparse. A few available studies have revealed the previously unexplored sequence space and heterogeneity of GSB-associated viruses [8,23]. It was also demonstrated that these phages are active and highly dynamic, and can play a significant role in the evolution of their hosts [8,23]. Here, we presented an analysis of viral sequences recovered from *C. clathratiforme* single-cell amplified genomes, further uncovering the genomic structure and repertoire of putative phages associated with these naturally occurring, highly abundant, sulfur-oxidizing bacteria. However, the relationships between recovered viral contigs and *C. clathratiforme* SAGs should still be interpreted with some caution, as there is a chance that some of these viral contigs might have originated from accidentally co-sorted free virus particles. Nevertheless, our study specifically revealed (i) new genetic information (Table S8) about the distribution (Figure 2) of the viral community associated with *C. clathratiforme*, (ii) the high level of lysogenization in the *C. clathratiforme* population (Table S4), (iii) the genomic potential of phages to contribute to biological sulfur cycling via viral-encoded AMGs (Table 1), and (iv) the dynamic nature of interactions between specific putative phages and *C. clathratiforme* (Table 2). Our results suggest that *C. clathratiforme* might be a host organism for *Microviridae* phages (Table 2, Table S4). Finally, we also suggest that *C. clathratiforme* is likely a “keystone” species in Lake Kirkilai (Figure 1), which represents a model gypsum karst lake ecosystem (EUR 28). Altogether, these findings contribute to an improved understanding of the ecological complexity of rare and unique environments.

The majority of viral gene and protein sequences determined in this study were classified as “unknown” and lacked detectable homologs in public sequence databases. In this regard, our findings are similar to those in Llorens-Marès et al. [8] and Berg et al. [23], which used bioinformatic approaches to reconstruct and analyze putative phage contigs and genomes associated with GSB from metagenomes. The researchers found low percentages of gene/genome sequence similarities between GSB-associated and other known viruses in these studies. Using comparative genomics analysis, they also observed that GSB-associated viral contigs clustered at a distance from other phages, potentially representing novel and separate viral lineages [8,23]. This is not unexpected, however, and such a high number of “unknowns” can be attributed to both vastly undersampled euxinic environments dominated by GSB [62] and the highly divergent nature of members of *Caudovirales*. The high diversity of viruses potentially infecting GSB may be associated with the highly specialized and endemic (ecotypes) distribution of GSB species, as well as to the modular structure of virus–host interactions [63], which in turn prevents cross-taxon genomic recombination between co-infecting phages resulting in genetically discrete populations [64]. Finally, the lack of identifiable genes in common between *C. clathratiforme* SAGs-associated viral contigs and those characterized in Llorens-Marès et al. [8] and Berg et al. [23], as well as the presence of the high number and diversity of spacers in the *C. clathratiforme* CRISPR loci (Table S9) with no matches to known viral sequences, suggests that the much broader diversity of GSB viruses awaits future discoveries.

In addition to the observed large number and high diversity of *C. clathratiforme* SAGs-associated viral contigs, our study also suggests coinfections in the *C. clathratiforme* population (average—7 viral contigs/SAG, median—6 viral contigs/SAG; Table S6). Even though recovered phage sequences may represent remnants of past infections, they are still an indication of the extensive virus–host interactions and increased eco-evolutionary pressure on both host bacteria and their phages, including the phage life cycle decision (e.g., lytic vs. lysogenic; [65]). The conversion from the lytic to lysogenic replication pathway may have significant implications for phage distribution within the host population [66,67]. In this study, we found that most of *C. clathratiforme* SAGs (87%) contained a putative prophage (Figure 2b), revealing a high level of lysogenization within the *C. clathratiforme* population. Similarly, Berg et al. [23] found that some of GSB populations can be persistently infected (throughout ~11 years) by the temperate phages at very high rates (~100%; [23]). Attempting to explain these observations, Berg and colleagues [23] proposed that the clonal composition of the host population, concomitant with a low rate of prophage induction, might promote the distribution of temperate phages within the population. Our findings of prophage prevalence in most of the *C. clathratiforme* SAGs (54/62), all of which have high (>99%) 16S rRNA gene nucleotide sequence similarity, agree with the hypothesis described in Berg et al. [23]. On the other hand, however, it has been shown that an increased level of co-infections may also promote the rate of genome integrations of temperate phages (life-cycle decision; [67,68]). The lysogenization rate of the host population can also be density-dependent (“Piggyback-the-winner” (PtW) hypothesis; [68]). Thus, the high level of *C. clathratiforme* lysogenization observed in our study can be explained by a combination of several factors, including the clonal composition [23] and high density (PtW strategy; [68]) of the host population (Figure 2, Table S2), as well as by the high levels of coinfections by the co-occurring phages (Table S6). From the phage perspective, lysogenization may provide a competitive advantage against other co-infecting phages (super-infection exclusion; e.g., [69] and references therein) and ensure more stable co-existence between the phage and its host on a temporal scale, such as during the periods of GSB blooms.

Numerous metagenomic surveys revealed that extremely diverse viral communities often exhibit species–rank abundance distribution [70]. These observations led to the development of the Bank model hypothesis [54], which states that at any given time, there is only a small subset of highly abundant viruses complemented by a large number of rare genotypes. This fraction of rare viruses is maintained at low densities until their suitable hosts reach abundances high enough to promote their replication, leading to changes in the dominant viral genotypes [54]. The *C. clathratiforme* SAGs-associated viral contigs showed rank–abundance distribution at the population level (Figure 2b) similar to that typically observed for entire viral communities, thus demonstrating the existence of the same principles proposed in the Bank model at a lower organizational level. Such distribution of putative *C. clathratiforme* phages may also imply that temperate phages, at least at the time of sampling and in the presence of clonal composition of the host population, were relatively more “successful” than most of the lytic phages. The observed distribution of putative *C. clathratiforme* phages (Figure 2b) also fits quite well with the distribution of CRISPR spacers in the *C. clathratiforme* genome (Table 2). For example, the highest number of spacers (Table 2) was observed against the putative phages that were relatively more abundant (found in more than 10% of SAGs, e.g., NODE_31, NODE_48, and NODE_113; Table S2). On the other hand, the CRISPR-mediated defense could have limited the more extensive distribution of these and other putative viruses (e.g., NODE_77, NODE_395, and NODE_396; Table 2) within the *C. clathratiforme* population. Collectively, however, these findings demonstrate the ongoing interactions between individual phages emerging from the large pool of *C. clathratiforme* viruses and its host, which eventually determine their distribution in a given environment.

Members of *Chlorobium* perform anoxygenic photosynthesis using reduced sulfur compounds (e.g., thiosulfate, sulfide, etc.) as electron donors to fixate carbon dioxide. The oxidation of sulfur can proceed via canonical Sox and/or Dsr pathways [71,72]. Genes involved in Sox (*soxYZ*) and Dsr (*dsrC*) pathways were previously identified in viral metagenomes [62], though they were absent in GSB-associated viral contigs described in Llorens–Marès et al. [8] and Berg et al. [23]. In addition to these already known AMGs, we identified thiosulfate sulfurtransferase (rhodanese; *TST*) (Table 2), which catalyzes the transfer of a sulfur atom from thiosulfate to thiophilic acceptors [72]. The thiosulfate sulfurtransferase, by breaking the S–S bond present in thiosulfate, may provide an alternative way of thiosulfate reduction to sulfur and sulfite [73], which subsequently can be used for the biosynthesis of Fe/S clusters, amino acids, various cofactors, redox reactions, and regulatory pathways [72]. Another identified AMG is the thioredoxin-dependent PAPS reductase (cysH; Table 2) involved in assimilatory pathways of sulfate reduction. These enzymes take part in the biosynthesis of sulfite by catalyzing the reduction of protein disulfide bonds in PAPS [74]. In addition to these, one ORF was found to encode the iron–sulfur cluster assembly protein (encoded by *iscA* gene; Table S8) known to take part in the recruitment of intracellular iron during the biogenesis of iron–sulfur clusters, which requires the coordinated incorporation of both iron and sulfur [75,76]. The Fe/S cluster assembly protein participates in electron transport, redox reactions, and regulatory processes. Thus, although the precise metabolic role of the AMGs mentioned above during infection remains unknown, it might be that *C. clathratiforme*-infecting phages utilize these enzymes to modulate sulfur-oxidation rates toward the increased energy production required for effective phage replication.

Some metagenomic datasets indicated that small single-stranded DNA (ssDNA) phages belonging to *Gokushovirinae* and a group of unclassified *Microviridae* are widely distributed in aquatic viral communities [70,77,78], suggesting that these microviruses can infect a broad range of host taxa. The recovery of the *Microviridae* genome (NODE_396; Table S2) and the detection of CRISPR spacers against this and other microviruses (Table 2) suggest that members of *Microviridae* can infect *C. clathratiforme*, and that there exists a proactive intracellular defense system to prevent these infections. Further studies, however, are needed to gain a more comprehensive understanding of interactions between *Microviridae* phages and *C. clathratiforme*.

## 5. Conclusions

A comprehensive understanding of viral activity in aquatic ecosystems remains limited by the lack of host-contextualized quantitative surveys of viral diversity. This study, using a single-cell sequencing approach, provides viral genomic data experimentally linked to specific ecologically relevant green sulfur bacteria, *C. clathratiforme*. We showed the existence of a large pool of diverse viruses that have not yet been cultivated and that are distinct from those in public databases and aquatic metagenomes, potentially representing new lineages. We also reported a high level of *C. clathratiforme* population lysogenization and the potential of phages to alter sulfur metabolism and cycling. We anticipate that new genomic information retrieved from *C. clathratiforme* SAGs will improve our knowledge about phage diversity associated with green sulfur bacteria, abundant in both euxinic waters and in rare habitats such as gypsum karst lakes.

## Figures and Tables

**Table 1:** Auxiliary metabolic genes identified in *C. clathratiforme* SAGs-associated viral contigs.

Metabolism	Pathway	Total AMGs	AMG KO ^a^	AMG KO Name
Carbohydrate metabolism	Pentose phosphate pathway	1	K07404	pgl; 6-phosphogluconolactonase
	Fructose and mannose metabolism	4	K01711	Gmd; GDPmannose 4,6-dehydratase
			K02377	fcl; GDP-L-fucose synthase
	Galactose metabolism	1	K01784	gale; UDP-glucose 4-epimerase
	Amino sugar and nucleotide sugar metabolism	7	K01709	rfbG; CDP-glucose 4,6-dehydratase
			K01711	Gmd; GDPmannose 4,6-dehydratase
			K02377	fcl; GDP-L-fucose synthase
			K13010	rfbE; perosamine synthetase
	C5-Branched dibasic acid and Butanoate metabolism	1	K01652	ilvB, ilvG, ilvI; acetolactate synthase I/II/III large subunit
Energy metabolism	Sulfur metabolism	2	K00390	cysH; thioredoxin-dependent phosphoadenosine phosphosulfate (PAPS) reductase
		2	K02439	TST; thiosulfate sulfurtransferase (rhodanese)
Amino acid metabolism	Alanine, aspartate and glutamate metabolism	1	K01953	asnB; asparagine synthase (glutamine-hydrolyzing)
	Glycine, serine and threonine metabolism	1	K00613	Glycine amidinotransferase
	Cysteine and methionine metabolism	3	K00558	dcm; DNA (cytosine-5)-methyltransferase
			K00789	metK; S-adenosylmethionine synthetase
	Valine, leucine, and isoleucine biosynthesis	1	K01652	ilvB, ilvG, ilvI; acetolactate synthase I/II/III large subunit
	Arginine and proline metabolism	1	K00613	Glycine amidinotransferase
	Cysteine and methionine metabolism		K00558	dcm; DNA (cytosine-5)-methyltransferase
Metabolism of cofactors and vitamins	Pantothenate and CoA biosynthesis	1	K01652	ilvB, ilvG, ilvI; acetolactate synthase I/II/III large subunit
	Porphyrin and chlorophyll metabolism	1	K04034	bchE; anaerobic magnesium-protoporphyrin IX monomethyl ester cyclase
	Ubiquinone and other terpenoid-quinone biosynthesis	2	K03183	ubiE; demethylmenaquinone methyltransferase/2-methoxy-6-polyprenyl-1,4-benzoquinol methylase
Unclassified	–	1	K02039	phoU; phosphate transport system protein

^a^ KO—KEGG Orthology database of molecular functions represented in terms of functional orthologs.

**Table 2:** Summary of CRISPR arrays identified in the consensus genome of *C. clathratiforme* and spacer sequence matches *C. clathratiforme* SAGs-associated viral contigs and viral sequences in publicly available repositories (accessed on March 2021).

CRISPR No. (Subfamily)	Array Length (bp)	Repeat Sequence (5′→3′)	Cas Proteins	Number of Spacers	Spacer No.	Viral Contig Match	ViralDB Match (e ≤ 10^−4^)	NCBI Accession No.
CRISPR1 (subfamily III-A)	2983	ATTATCTCCGACCTGACATATCAAAAGGGATTACGAC	Cas1, Cas2, Cas6, Cas10, Csm2, Csm3, Csm4, Csm5, RT, DExK	40	13	NODE_31	–	–
					35	NODE_77	–	–
					37	NODE_31	–	–
			
			
			
			
			
			
CRISPR2 (subfamily I-C)	2078	GTCGCGCCCCCTGCGGGCGCGTGGATTGAAAC	–	31	1	NODE_31	–	–
					4	NODE_113	–	–
					5	NODE_396	Gokushovirus WZ-2015a	KT264813.1
					16	–	*Microviridae* ctec913	MH617588.1
					18	NODE_113	–	–
					19	NODE_113	–	–
CRISPR3 (subfamily Unknown)	5829	GTTTCAATCCGCTATGCGTGCAATAAGATATGATG	Cas 1, Cas2	81	52	NODE_31	–	–
					54	NODE_48	Erythrobacter phage vB_EliS_R6L	KY006853.1
					62	NODE_395	–	–
					66	NODE_31	–	–
					73	NODE_48	–	–

## Data Availability

The 16S rDNA sequences obtained using the 515F-Y/926R prokaryotic primers set and single cell sequences presented in this article have been submitted to NCBI and are available under BioProject accession number PRJNA562654.

## Supplementary Materials

The following are available online at https://www.mdpi.com/article/10.3390/genes12060886/s1, Figure S1: Rarefaction plots of the four samples taken at the different depths in Lake Kirkilai (Lithuania), Figure S2: Shannon index of OTU diversity and Simpson evenness value at each depth Lake Kirkilai (Lithuania), Figure S3: Neighbor-joining tree reconstruction based on the full length 16S rDNA multiple sequence alignment generated from MAFFT v7. The numbers at nodes refer to bootstrap support values (number of resampling: 1000). The scale bar equals 0.4 substitutions per site. Figure S4: Proteomic tree of 2769 prokaryotic dsDNA viruses including 82 viral contigs found in this study. The dendrogram represents the proteome-wide similarities between *C. clathratiforme* SAGs-associated viral contigs (red start symbol and red branches) and other prokaryotic viruses (black branches). Branch lengths are logarithmically scaled from the root of the entire proteomic tree. Numbers represent genomic similarity scores (S_G_) retrieved from normalized tBLASTx bit scores computed from the pairwise viral sequence comparisons. Rings represent viral (inner ring) and host (outer ring) taxonomic classifications. The proteomic tree was generated with ViPTree software v1.9, Figure S5: Neighbor-joining tree reconstructions based on the different marker genes identified in *C. clathratiforme* SAGs-associated viral contigs. Multiple sequence alignments were generated using MAFFT v7. The numbers at nodes refer to bootstrap support values expressed as percentages (number of resampling: 1000), Table S1: Environmental characteristics of the four sampling sites in Lake Kirkilai (Lithuania), Table S2: Taxonomic classification of the most abundant OTUs (>1% or more of the total reads) throughout the water column of the Lake Kirkilai (Lithuania), Table S3: Single-cell genome assembly statistics, Table S4: General characteristics, taxonomic classification and distribution of *C. clathratiforme* SAGs-associated viral contigs, Table S5: BLASTn search results against IMG/VR dataset (Ecosystem category: Aquatic; accessed June 2021; database version 5.1), Table S6: Viral abundance estimates normalized as fragments per kilobase per mapped million reads (FPKM) for each individual *C. clathratiforme* SAG, Table S7: Genome annotations of *C. clathratiforme* SAGs-associated viral contigs, Table S8: Similarity matrix of *Microviridae* phage genomes (RefSeq DB; accessed March 2021) generated using VIRIDIC software, Table S9: Predicted protein coding genes in *C. clathratiforme*-associated phage sequences, Table S10: List of CRISPR spacer sequences and spacer sequence matches in *C. clathratiforme* SAGs-associated viral contigs and other known phages identified in the consensus *C. clathratiforme* genome. File S1. Genome annotation file of *C. clathratiforme*.
