# Supplementary material for: Exploring Viral Diversity in a Gypsum Karst Lake Ecosystem Using Targeted Single-Cell Genomics
Source: Genes (Basel). 2021 Jun 8;12(6):886. doi: 10.3390/genes12060886 (PMC8226683; doi:10.3390/genes12060886)
Supplement: Supplementary file 1 [file genes-12-00886-s001.zip › genes-1229203-SI/SI_Figures.pdf]

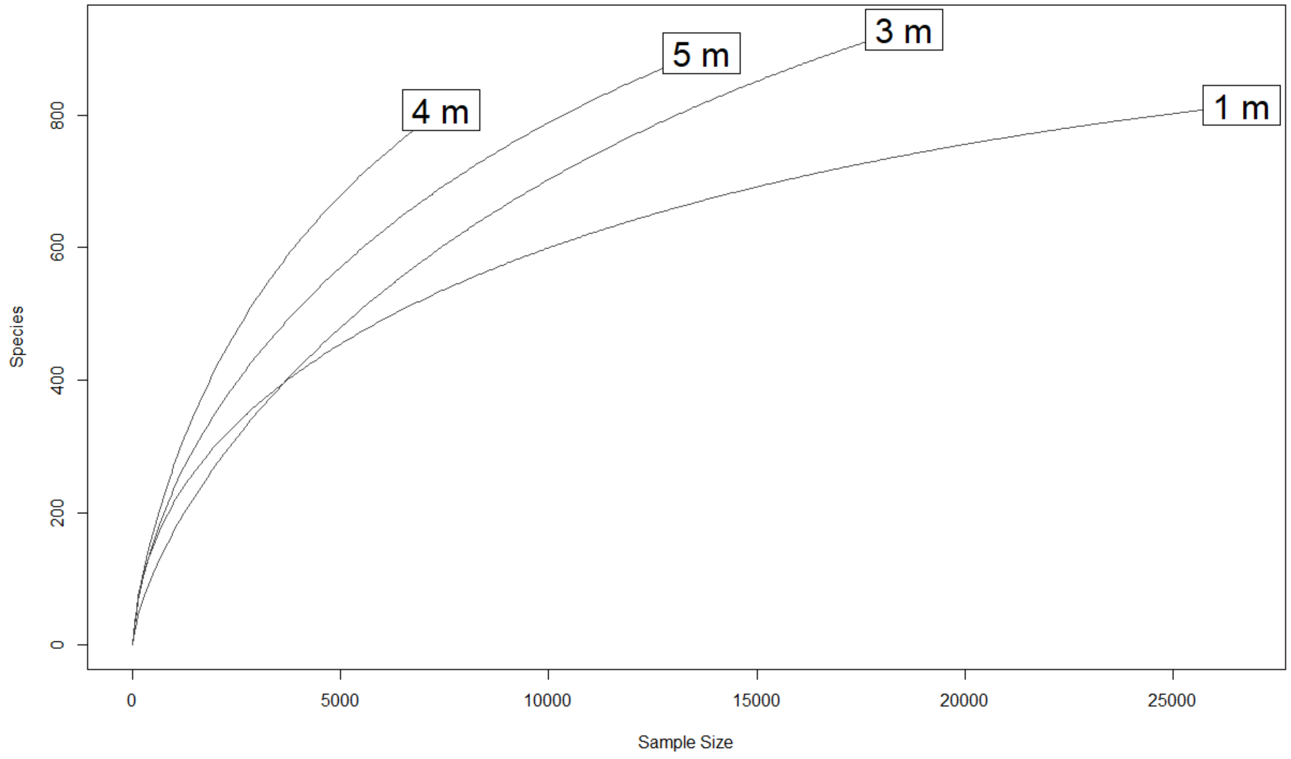

Figure S1. Rarefaction plots of the four samples taken at the different depths in Lake Kirkilai (Lithuania). Sample size refers to the number of randomly subsampled read pairs.

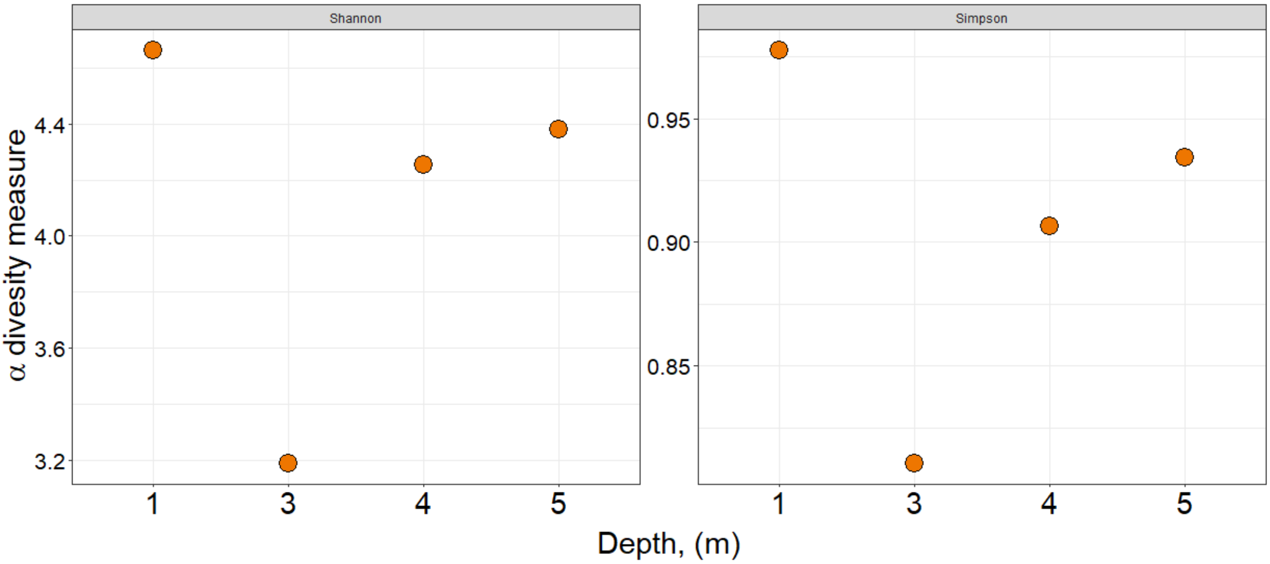

Figure S2. Shannon index of OTU diversity and Simpson evenness value at each depth Lake Kirkilai (Lithuania).

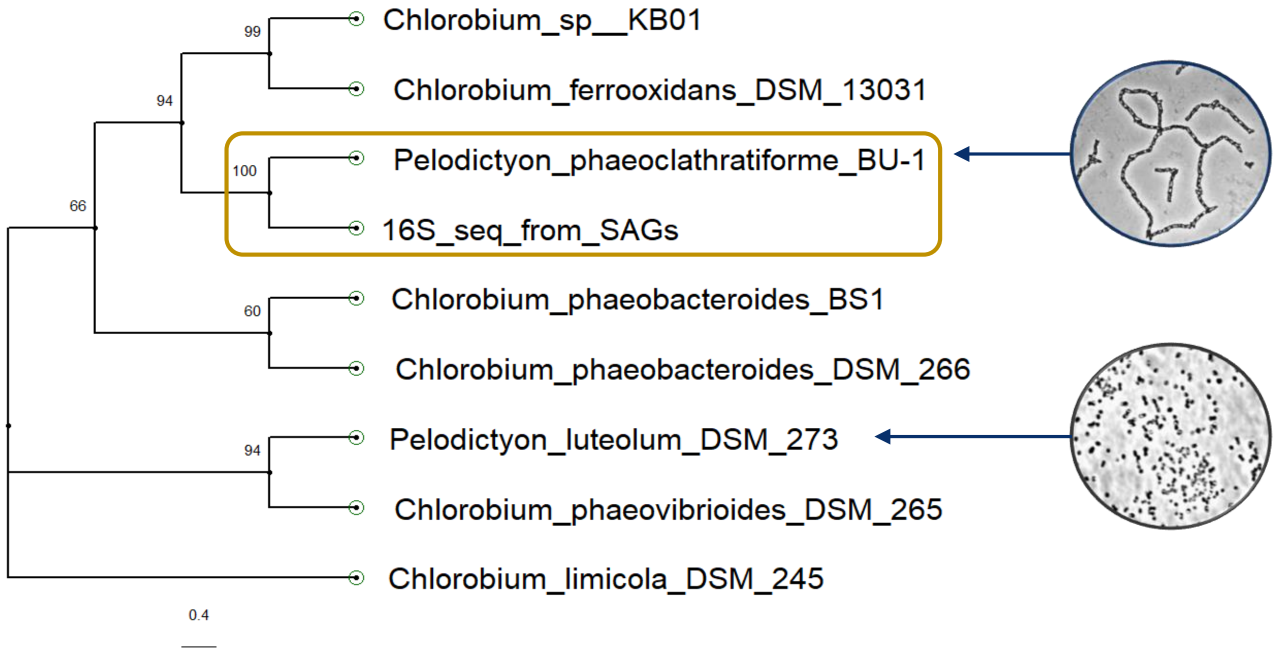

Figure S3. Neighbor-joining tree reconstruction based on the full length 16S rDNA multiple sequence alignment generated from MAFFT v7. The numbers at nodes refer to bootstrap support values (number of resampling: 1000). The scale bar equals 0.4 substitutions per site.

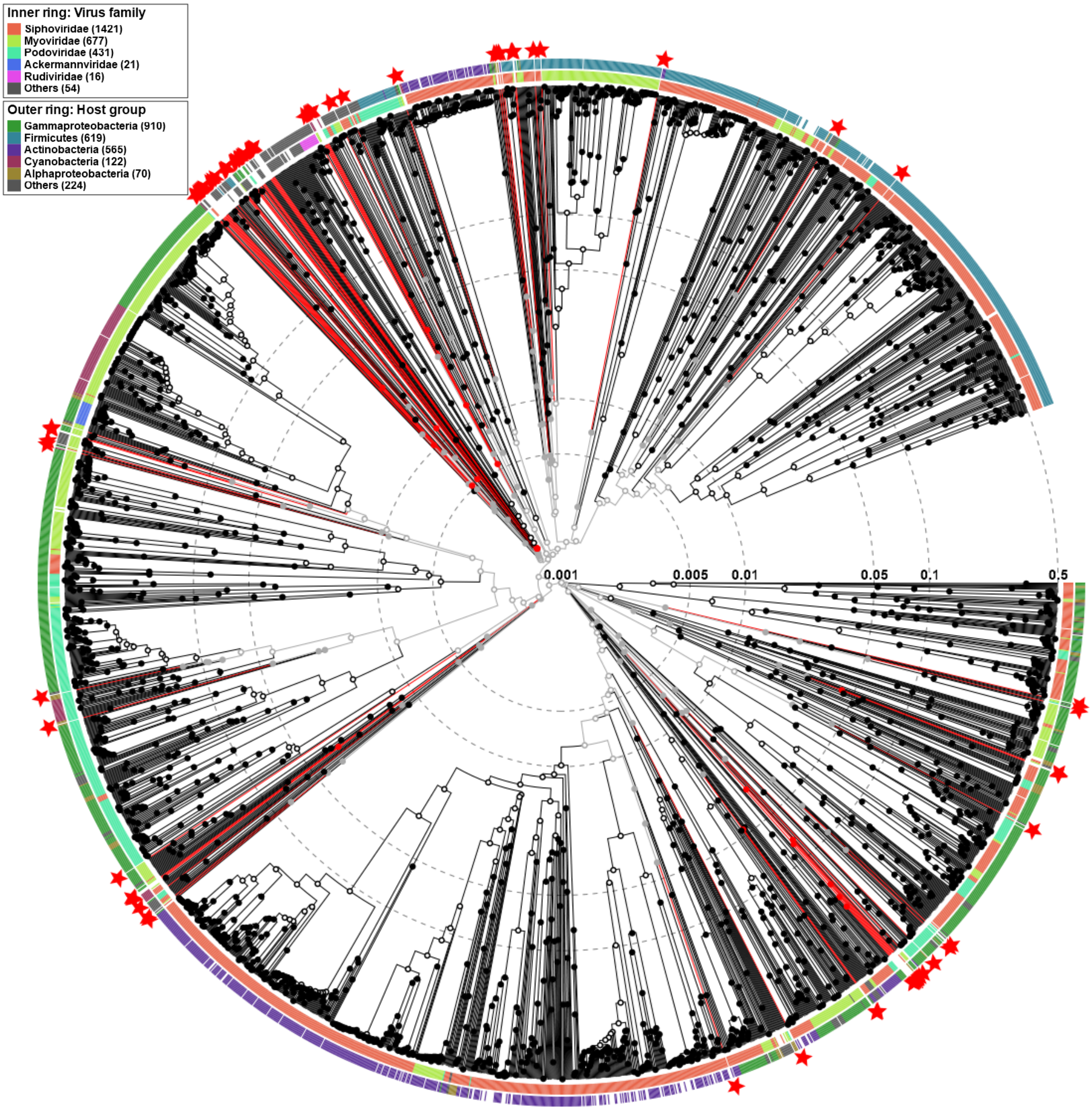

Figure S4. Proteomic tree of 2769 prokaryotic dsDNA viruses including 82 viral contigs found in this study. The dendrogram represents the genome-wide protein similarities between *C. clathratiforme* SAGs-associated viral contigs (red start symbol and red branches) and other prokaryotic viruses (black branches). Branch lengths are logarithmically scaled from the root of the entire proteomic tree. Numbers represent genomic similarity scores (S_G) retrieved from normalized tBLASTx bit scores computed from the pairwise viral sequence comparisons. Rings represent viral (inner ring) and host (outer ring) taxonomic classifications. The proteomic tree was generated with ViPTree software v1.9.

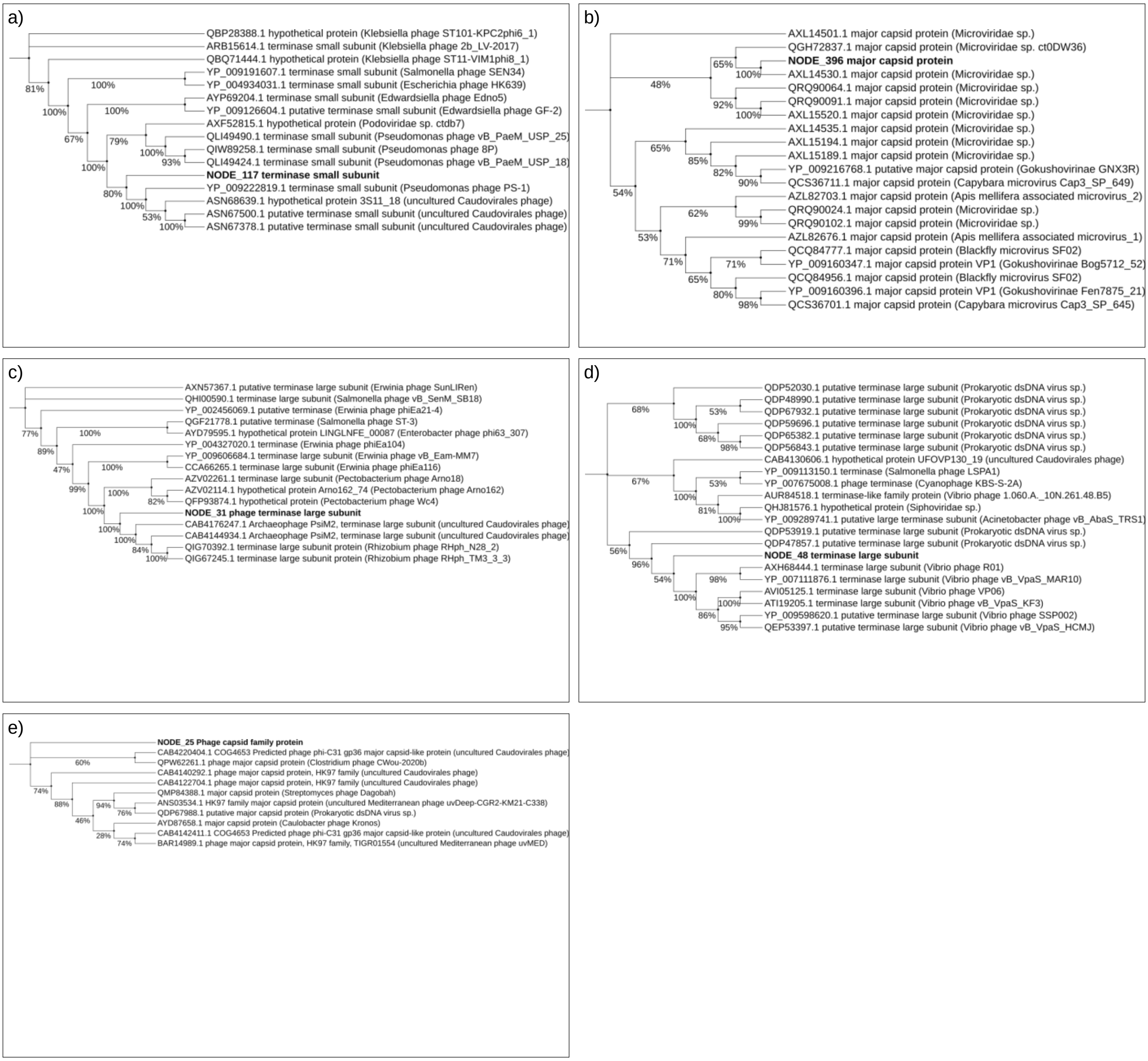

Figure S5. Neighbor-joining tree reconstructions based on the different marker genes identified in *C. clathratiforme* SAGs-associated viral contigs. Multiple sequence alignments were generated using MAFFT v7. The numbers at nodes refer to bootstrap support values expressed as percentages (number of resampling: 1000).
