# Supplementary material for: Exploring Viral Diversity in a Gypsum Karst Lake Ecosystem Using Targeted Single-Cell Genomics
Source: Genes (Basel). 2021 Jun 8;12(6):886. doi: 10.3390/genes12060886 (PMC8226683; doi:10.3390/genes12060886)
Supplement: Supplementary file 1 [file genes-12-00886-s001.zip › genes-1229203-SI/Table_S1_ENV.pdf]

genes

MDPI

Table S1. Environmental characteristics of the four sampling sites in Lake Kirkilai (Lithuania)

Parameter	Depth			
	1 m	3 m	4 m	5 m
Temperature, °C	22.60	19.60	15.70	12.50
pH	8.03	7.17	7.01	6.22
Conductivity, µS cm^{-1}	1468	1959	2270	2340
Salinity	0.70	1.00	1.10	1.20
O_2, mg L^{-1}	6.27	0.98	0.00	0.00
N_{TOTAL}, mg L^{-1}	1.38	1.48	1.64	2.38
NO_3, mg N L^{-1}	0.62	<0.01	<0.01	<0.01
NH_4, mg N L^{-1}	0.16	0.08	0.43	1.03
P_{TOTAL}, mg L^{-1}	0.02	0.02	0.03	0.06
PO_4, mg P L^{-1}	<0.001	0.001	0.010	0.026
N:P	31	33	25	18
